# Utility of Postoperative Thyroid Hormone Levels in Predicting the Diagnosis of Adult Growth Hormone Deficiency in Patients Undergoing Surgery for Nonfunctioning Pituitary Neuroendocrine Tumor: A Pilot Study

**DOI:** 10.1155/ije/8842344

**Published:** 2026-06-09

**Authors:** Yuki Tsujimoto, Kenji Yamashiro, Yuki Suzuki, Yui Watanabe, Yudo Ishii, Rimei Nishimura

**Affiliations:** ^1^ Department of Internal Medicine, Division of Diabetes, Metabolism, and Endocrinology, Jikei University School of Medicine, Tokyo, Japan, jikei.ac.jp; ^2^ Department of Neurosurgery, Jikei University School of Medicine, Tokyo, Japan, jikei.ac.jp

**Keywords:** adult growth hormone deficiency, free thyroxine, free triiodothyronine, nonfunctioning pituitary neuroendocrine tumor, thyroid hormone

## Abstract

**Background:**

Adult growth hormone deficiency (AGHD) is often diagnosed after surgery for nonfunctioning pituitary neuroendocrine tumor (NF‐PitNET). While a growth hormone stimulation test (GHST) is required to establish its diagnosis, it is associated with a large burden on patients and healthcare providers alike. Currently, the recommended indicators for the diagnosis of AGHD include the IGF‐1 standard deviation score (IGF‐1SDS) from a random blood sample and the number of deficiencies in pituitary hormones other than GH; however, there are also some challenges associated with these indicators. Therefore, to predict the need for GHST, this study focused on the interrelationship between GH and pituitary hormones to investigate whether postoperative thyroid‐stimulating hormone (TSH), free triiodothyronine (FT3), and free thyroxine (FT4) levels measured before GHST could represent novel predictors of AGHD.

**Methods:**

Among 166 patients who underwent GHST following surgical treatment for NF‐PitNET, 60 patients who were diagnosed with NF‐PitNET based on clinical and pathological criteria were included in this analysis. Exclusion criteria were as follows: missing data (*n* = 16), data collection outside the specified timeframe (*n* = 4), positive thyroid autoantibodies (*n* = 2), and oral levothyroxine administration during GHST (*n* = 3). Clinical parameters of the 35 patients were compared between the AGHD group (*n* = 11) and the non‐AGHD group (*n* = 24) using Fisher’s exact test and Mann–Whitney *U* test.

**Results:**

Patients with AGHD showed significantly lower postoperative levels of FT3 and FT4 prior to GHST, with median values of 2.10 pg/mL (1.97–2.34) and 0.98 ng/dL (0.77–1.19), respectively, than those without postoperative AGHD, who showed median levels of 2.45 pg/mL (2.28–2.67) and 1.18 ng/dL (1.03–1.26) (*p* < 0.001 and *p* = 0.008, respectively). In contrast, no significant difference in TSH levels was observed between the two groups. Receiver operating characteristic (ROC) analysis identified the optimal cutoff values for predicting AGHD as 2.125 pg/mL for FT3 and 1.010 ng/dL for FT4.

**Conclusion:**

These findings suggest the potential utility of FT3 and FT4 as novel predictors for the diagnosis of postoperative AGHD.

## 1. Introduction

Adult growth hormone deficiency (AGHD) is associated with alterations in body composition, such as increased body fat and reduced muscle mass, dyslipidemia, osteoporosis, and metabolic dysfunction‐associated steatotic liver disease (MASLD) [1, 2]. Furthermore, it is known to be associated with reduced quality of life (QOL) and increased risk of cardiovascular events [1, 2]. Among these complications, body composition abnormalities, osteoporosis, MASLD, and reduced QOL can be improved with GH replacement therapy [3, 4]. Thus, AGHD is an important disease that requires appropriate diagnosis and treatment. GH secretion is characterized by a pulsatile pattern with diurnal variability and is influenced by dietary factors [5]. Consequently, diagnosing AGHD based on a single blood sample is challenging, necessitating the use of an adjunctive GH stimulation test (GHST), such as the insulin tolerance test and/or the GH‐releasing peptide‐2 (GHRP2) test [6].

Major causes of AGHD include treatments for suprasellar tumors, cerebrovascular disease, inflammatory disease, and traumatic injury [7]. Among these, a nonfunctioning pituitary neuroendocrine tumor (NF‐PitNET) is the most common [8, 9]. Some studies comparing the incidence of AGHD before and after surgery have shown a decrease in its incidence, whereas others have shown an increase in its incidence after surgery, suggesting no consistent effect of surgery on AGHD [10–12].

Postoperative GHST is required to examine whether patients with NF‐PitNET have AGHD after surgery. However, GHST represents a large burden on patients and healthcare providers alike, making it unrealistic to perform it on all patients who are undergoing surgery for NF‐PitNET. Thus, efforts have been made to identify predictive factors that would prove useful in determining whether GHST is warranted to facilitate the diagnosis of AGHD. Indeed, the standard deviation score for insulin‐like growth factor 1 (IGF‐1SDS) and the number of deficits involving pituitary hormones other than GH may facilitate the diagnosis of AGHD in patients with pituitary tumors who are deemed at risk of AGHD [13]. However, these indicators are not without their limitations: IGF‐1SDS has been shown to predict the diagnosis of AGHD effectively, though its accuracy is limited in patients older than 60 years [14]; again, while the presence of three or more pituitary hormone deficits has been shown to be highly associated with the diagnosis of AGHD [15], the presence of two or fewer hormone deficits requires additional tests to establish its diagnosis. In light of these observations, it is concluded that the predictive accuracy of the IGF‐1SDS is unclear, and the number of hormone deficits present may only prove useful in predicting the diagnosis of AGHD in a limited population of patients. Thus, there remains a significant need for predictors of AGHD in a larger population of patients at risk of AGHD.

With a focus on the relationship between GH and thyroid hormones, as typically shown in GH replacement therapy leading to changes in thyroid hormone levels [16], this study investigated whether thyroid hormones might represent potential predictors of AGHD. Specifically, this study focused on patients with NF‐PitNET, the most common cause of AGHD, to investigate how thyroid hormones and AGHD might be associated, whether these patients might be shown to have AGHD after surgery for NF‐PitNET, and whether their FT3 and FT4 levels, measured after surgery for NF‐PitNET and before GHST, might allow the prediction of AGHD.

## 2. Methods

### 2.1. Patients and Evaluations

Medical records were systematically reviewed of patients who underwent pituitary function testing at Jikei University Hospital (Tokyo, Japan) between January 2017 and March 2023. A total of 166 patients were initially identified from the institutional database. The present study focused on patients who had undergone endoscopic transsphenoidal surgery and were diagnosed with NF‐PitNET based on their clinical presentation and histopathological findings. Patients were required to have GHST at least 12 weeks postoperatively to allow recovery from surgical stress and stabilization of pituitary function [17]. This time interval was chosen to minimize the acute effects of surgery on GH axis assessment. Of the 166 patients initially identified, 60 patients met these criteria for NF‐PitNET diagnosis and postoperative GHST timing.

Exclusion criteria were as follows: missing data (*n* = 16), data collection outside the specified timeframe (*n* = 4), positive thyroid autoantibodies (*n* = 2), and oral levothyroxine administration during GHST (*n* = 3). After applying these exclusion criteria, 35 patients were included in the final analysis.

The GHST consists of an insulin tolerance test and a GHRP2 stimulating test. The insulin tolerance test involved administering fast‐acting insulin at a dose of 0.1–0.15 U/kg (or less or more depending on the patient’s condition and at the discretion of the attending physician), blood sampling at 30‐min intervals from 0 to 90 min after dosing, and measuring blood glucose and GH levels, where the response was construed as valid when it involved a decrease in blood glucose to less than 45 mg/dL or less than half the glucose value measured at baseline; as it decreased when it involved peak GH levels less than 1.8 ng/mL, and as undeterminable when it involved neither [18]. The GHRP2 stimulating test, which is routinely performed in Japan and offers comparable accuracy to that of insulin hypoglycemia testing [19], involves administering 100 μg intravenously of GHRP2, blood sampling at 15 min intervals from 0 to 60 min after dosing, and measuring GH levels at these points, where the response is construed as decreased when it is associated with peak GH levels less than 9.0 ng/mL [19, 20].

**FIGURE 1:**
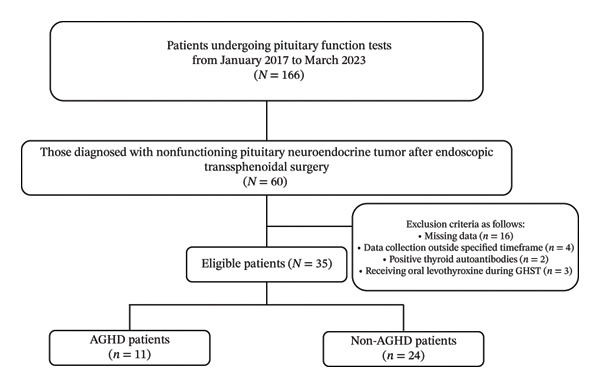
Patient characteristics.

Patients were diagnosed with AGHD if, according to the Japanese guidelines, they exhibited a decreased GH response in both the insulin tolerance test and the GHRP2 stimulating test or if they showed a decreased response in either test and had a deficiency of one or more pituitary hormones in addition to GH [18]. Patients were categorized into AGHD and non‐AGHD groups. Those diagnosed with AGHD as described above were classified into the AGHD group, while all patients who did not exhibit a decreased GH response despite adequate hypoglycemia in the insulin tolerance test and did not show a decreased response in the GHRP2 test were classified into the non‐AGHD group. Consequently, a total of 35 patients (AGHD, *n* = 11; non‐AGHD, *n* = 24) were ultimately included in the analysis (Figure 1). For all patients included in the final analysis, the following postoperative data were obtained from medical records: age, sex, medical history (diabetes mellitus, hypertension, and dyslipidemia), maximum pituitary tumor diameter, height, body weight, body mass index (BMI), liver function tests including aspartate aminotransferase (AST) and alanine transaminase (ALT), the FIB‐4 index, estimated glomerular filtration rate (eGFR), and basal pituitary hormone levels including GH, insulin‐like growth factor‐1 (IGF‐1), thyroid‐stimulating hormone (TSH), free triiodothyronine (FT3), free thyroxine (FT4), adrenocorticotropic hormone (ACTH), cortisol, luteinizing hormone (LH), and follicle‐stimulating hormone (FSH) measured during the postoperative period. Additionally, preoperative thyroid function parameters (TSH, FT3, and FT4) were analyzed in patients when available.

To minimize the potential confounding effects of surgical stress on thyroid hormone levels, thyroid function parameters used for analysis were obtained from blood samples collected at least 4 weeks postoperatively [17] and prior to GHST, allowing these parameters to serve as potential predictors of GHST outcomes.

### 2.2. Assays

GH levels were determined via the ECLIA method (Elecsys hGH reagent; Roche, Switzerland). IGF‐1 levels were determined via the IRMA method (IGF‐1 IRMA “Daiichi”, Fujirebio, Japan), and IGF‐1SDS were calculated after adjustment for age and sex among Japanese individuals [21]. The basal serum TSH, FT3, and FT4 levels were determined via the CLEIA method with an AIA‐CL 2400 (Tosoh, Japan) and were 0.61–4.23 µIU/mL, 2.36–5.00 pg/mL, and 0.88–1.67 ng/dL, respectively.

### 2.3. Statistical Analysis

All the statistical analyses were performed via SPSS ver. 25.0 (SPSS Inc., Chicago, IL, USA; https://www.spss.com).

All continuous variables are presented as medians with interquartile ranges (25th–75th percentiles). Differences between two groups were assessed for statistical significance using the Mann–Whitney *U* test. Categorical variables were compared using Fisher’s exact test. Receiver operating characteristic (ROC) analysis was performed to determine the cutoff values of FT3 and FT4 for distinguishing AGHD from non‐AGHD. A *p* value of < 0.05 was considered to indicate statistical significance.

This study was approved by the Ethics Committee of Jikei University School of Medicine (IRB approval number: 33‐196 [10813]) and conducted in accordance with the Declaration of Helsinki and its amendments.

## 3. Results

Overall, the patients were a median age of 55.0 years (49.0–61.0) and had a BMI of 24.3 kg/m^2^ (22.9–27.5). The median intervals from surgery to postoperative GHST and from surgery to thyroid hormone measurement were 19.9 weeks (16.0–25.4) and 12.7 weeks (7.9–15.7), respectively. The median maximum tumor diameter of the NF‐PitNETs was 23.0 mm (20.0–29.0). Regarding hormone replacement therapy, hydrocortisone replacement was administered to two patients in the non‐AGHD group and one patient in the AGHD group. Moreover, no patients in either group required replacement therapy for gonadal or thyroid dysfunction.

A comparison between the non‐AGHD and AGHD groups revealed no significant differences in age, BMI, or time from surgery to postoperative GHST. There were no significant differences in the prevalence of diabetes mellitus or hypertension between the two groups. The maximum tumor diameter was significantly larger in the AGHD group than in the non‐AGHD group (26.0 (23.0–35.0) mm vs. 21.5 (19.0–25.8) mm, *p* = 0.012). Additionally, the prevalence of dyslipidemia was significantly higher in the non‐AGHD group than in the AGHD group (87.5% vs. 54.5%, *p* = 0.031) (Table 1). The biochemical data are presented (Supporting Table S1).

**TABLE 1:** Comparison of patient profiles between non‐AGHD patients and AGHD patients.

	**Overall (*n* = 35)**	**AGHD (*n* = 11)**	**Non-AGHD (*n* = 24)**	**p** **value**

Age, years	55.0 (49.0–61.0)	55.0 (49.0–60.0)	55.0 (49.3–62.5)	0.958
Male/Female	22/13	9/2	13/11	
Maximum tumor diameter, mm	23.0 (20.0–29.0)	26.0 (23.0–35.0)	21.5 (19.0–25.8)	0.012^∗^
Body mass index, kg/m^2^	24.3 (22.9–27.5)	26.7 (22.9–29.2)	24.0 (23.0–25.8)	0.163
Diabetes mellitus, *n* (%)	4 (11.4)	2 (18.2)	2 (8.3)	0.395
Hypertension, *n* (%)	24 (68.6)	7 (63.6)	17 (70.8)	0.670
Dyslipidemia, *n* (%)	27 (77.1)	6 (54.5)	21 (87.5)	0.031^∗^
Time from surgery to postoperative GHST, weeks	19.9 (16.0–25.4)	20.0 (15.9–25.9)	19.7 (16.3–25.3)	1.000
Time from surgery to thyroid hormone measurement, weeks	12.7 (7.9–15.7)	9.9 (6.7–14.3)	13.1 (8.5–16.5)	0.334

*Note:* The data are expressed as medians (25th–75th percentiles). The Mann‒Whitney test or Fisher’s exact test was used for comparisons between non‐AGHD patients and AGHD patients.

Abbreviation: GHST = growth hormone stimulation test.

^∗^ *p* < 0.05.

Comparison of endocrinological parameters related to GH revealed that the AGHD group exhibited significantly lower values than the non‐AGHD group. IGF‐1 levels were 86.0 (66.0–98.0) ng/mL vs. 116.5 (84.8–129.8) ng/mL (*p* = 0.006), and IGF‐1 SDS was −1.898 (−2.751 to −1.358) vs. −0.744 (−1.625 to −0.466) (*p* < 0.001). Additionally, basal levels of LH and FSH were significantly lower in the AGHD group compared to the non‐AGHD group (*p* = 0.005 and *p* = 0.012, respectively). Interestingly, while TSH levels did not differ between the two groups (1.28 (0.95–2.15) μIU/mL vs. 1.69 (1.02–2.17) μIU/mL, *p* = 0.563), FT3 and FT4 values were significantly lower in the AGHD group compared to the non‐AGHD group. FT3 levels were 2.10 (1.97–2.34) pg/mL vs. 2.45 (2.28–2.67) pg/mL (*p* < 0.001), and FT4 levels were 0.98 (0.77–1.19) ng/dL vs. 1.18 (1.03–1.26) ng/dL (*p* = 0.008) (Table 2). Furthermore, analysis of available preoperative thyroid function data revealed that among TSH, FT3, and FT4, only FT4 levels were significantly lower in the AGHD group compared to the non‐AGHD group (0.91 (0.79–1.03) ng/dL vs. 1.15 (0.97–1.26) ng/dL, *p* = 0.031) (Table 3).

**TABLE 2:** Comparison of the endocrinological data between non‐AGHD and AGHD patients.

	**Overall (*n* = 35)**	**AGHD (*n* = 11)**	**Non-AGHD (*n* = 24)**	**p** **value**

GH, ng/mL	0.16 (0.06–0.44)	0.11 (0.05–0.21)	0.21 (0.07–0.59)	0.163
IGF‐1, ng/mL	101.0 (78.0–125.0)	86.0 (66.0–98.0)	116.5 (84.8–129.8)	0.006^∗^
IGF‐1 SD score	−1.026 (−1.902 to −0.678)	−1.898 (−2.751 to −1.358)	−0.744 (−1.625 to −0.466)	< 0.001^∗^
TSH, μIU/mL	1.64 (0.98–2.15)	1.28 (0.95–2.15)	1.69 (1.02–2.17)	0.563
FT3, pg/mL	2.37 (2.19–2.58)	2.10 (1.97–2.34)	2.45 (2.28–2.67)	< 0.001^∗^
FT4, ng/dL	1.13 (0.99–1.26)	0.98 (0.77–1.19)	1.18 (1.03–1.26)	0.008^∗^
ACTH, pg/mL	13.2 (8.9–34.5)	10.0 (5.6–48.7)	20.4 (9.9–33.5)	0.390
Cortisol, μg/dL	9.69 (6.89–13.90)	9.75 (9.05–18.70)	9.52 (5.88–13.10)	0.211
LH, mIU/mL	5.00 (2.90–7.30)	2.90 (2.40–5.30)	6.50 (4.03–12.95)	0.005^∗^
FSH, mIU/mL	6.00 (4.20–11.10)	4.90 (3.00–6.00)	6.60 (4.88–28.95)	0.012^∗^

*Note:* The data are expressed as medians (25th–75th percentiles). The Mann‒Whitney test was used for comparisons between non‐AGHD patients and AGHD patients. ACTH, adrenocorticotropic hormone; FT3, free triiodothyronine; FT4, free thyroxine; IGF‐1, insulin‐like growth factor‐1.

Abbreviations: FSH = follicle‐stimulating hormone, GH = growth hormone, LH = luteinizing hormone, TSH = thyroid‐stimulating hormone.

^∗^ *p* < 0.05.

**TABLE 3:** Comparison of preoperative endocrinology data between non‐AGHD patients and AGHD patients.

	**Overall**	**AGHD**	**Non-AGHD**	**p** **value**

TSH, μIU/mL	1.44 (0.82–2.03)	1.64 (0.74–2.05)	1.44 (0.83–1.75)	0.897
FT3, pg/mL	2.25 (2.09–2.43)	2.15 (2.07–2.29)	2.35 (2.09–2.57)	0.119
FT4, ng/dL	1.05 (0.86–1.25)	0.91 (0.79–1.03)	1.15 (0.97–1.26)	0.031^∗^

*Note:* The data are expressed as medians (25th–75th percentiles). The Mann‒Whitney test was used for comparisons between non‐AGHD patients and AGHD patients. Missing data: TSH (*n* = 1), FT3 (*n* = 3), FT4 (*n* = 2). In the AGHD group, one patient had missing data for all three parameters (TSH, FT3, and FT4). In the non‐AGHD group, FT3 was missing in 2 patients and FT4 in 1 patient.

^∗^ *p* < 0.05.

**FIGURE 2:**
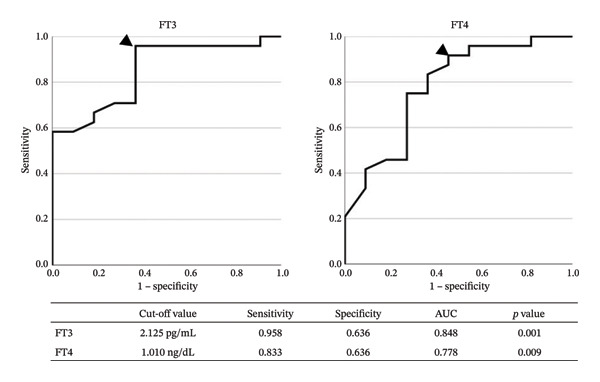
Cutoff values of FT3 and FT4 for predicting the diagnosis of postoperative adult growth hormone deficiency.

**FIGURE 3:**
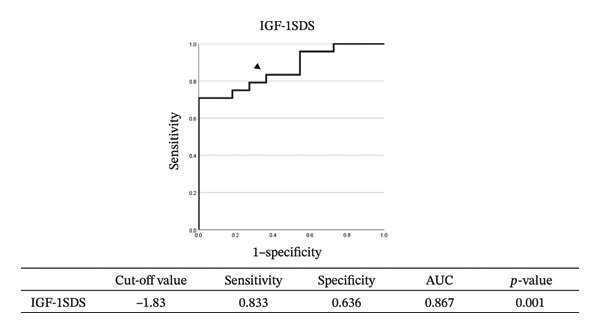
Cutoff values of the IGF‐1SDS for predicting the diagnosis of postoperative adult growth hormone deficiency.

Finally, the cutoff values for FT3 and FT4 for the prediction of AGHD were identified by ROC analysis as 2.125 pg/mL (sensitivity, 0.958; specificity, 0.636; area under the curve [AUC], 0.848; *p* = 0.001) and 1.010 ng/dL (sensitivity, 0.833; specificity, 0.636; AUC, 0.778; *p* = 0.009), respectively (Figure 2). The cutoff value for IGF‐1SDS in the prediction of AGHD was identified by ROC analysis as −1.83 (sensitivity, 0.833; specificity, 0.636; AUC, 0.867; *p* = 0.001) (Figure 3).

## 4. Discussion

The study results demonstrated that individuals diagnosed with AGHD based on the GHST after surgery for NF‐PitNET had lower postoperative FT3 and FT4 levels than those diagnosed with non‐AGHD, despite no significant difference in postoperative TSH levels. Furthermore, the optimal cutoff values for predicting AGHD based on thyroid hormones were determined to be 2.125 pg/mL for FT3 (sensitivity = 0.958, specificity = 0.636) and 1.010 ng/dL for FT4 (sensitivity = 0.833, specificity = 0.636). These results indicate that the predictive accuracy of these thyroid hormone parameters is comparable to that of IGF‐1SDS, a well‐established predictor of AGHD; specifically, FT3 demonstrated superior sensitivity, while FT4 showed sensitivity and specificity comparable to those of IGF‐1SDS. AGHD is known to occur with greater frequency in the presence of deficiencies in other pituitary hormones in addition to GH [13]. In the present study, the FT4 cutoff value was within the lower half of the reference range, suggesting that even when FT4 levels remain within the reference range, the possibility of AGHD cannot be excluded.

GH and other pituitary hormones are known to interact with each other, and FT3 and FT4 levels were shown in an earlier study to increase and decrease, respectively, with GH replacement therapy [16]. Furthermore, there have been reports indicating a positive correlation between FT3 and GH, as well as IGF‐1SDS, in patients with NF‐PitNET [22]. A comparison between patients with NF‐PitNET and those with acromegaly, whose GH is known to increase beyond its physiological range, revealed that FT3 levels were greater in patients with acromegaly [23]. On the other hand, the postoperative FT3 and FT4 levels of individuals with acromegaly were significantly lower and greater than their preoperative FT3 and FT4 levels, respectively, as their postoperative GH levels decreased [24]. These findings suggest that FT3 levels increase as GH levels increase, whereas FT3 levels decrease as GH levels decrease. It has been reported that GH promotes the conversion of thyroxine (T4) to triiodothyronine (T3) by promoting the activity of deiodinase type 2, an enzyme that deiodinates the inner ring of T4, thereby promoting the conversion of T4 to T3 and from T3 to 3,3′‐diiodothyronine [24, 25]. Thus, the postoperative FT3 levels, which were lower in the AGHD group than in the non‐AGHD group in this study, could be accounted for by a decrease in deiodinase type 2 activity associated with GH deficiency leading to a decrease in the conversion of T4 to T3.

However, it is important to consider the potential influence of surgical invasiveness on the study results, which demonstrated a postoperative decrease in both FT3 and FT4 levels. Indeed, a previous study demonstrated that NF‐PitNET itself could lead to central pituitary dysfunction, affecting as many as 34%–47% of patients before surgery and 34%–57% of patients after surgery [10, 11]. Previous studies have also shown that larger tumor size is associated with a higher incidence of postoperative pituitary hormone deficiencies [26, 27]. Consistent with these findings, patients in the AGHD group in the present study had significantly larger tumor sizes. Furthermore, LH and FSH levels were significantly lower in the AGHD group, suggesting more extensive pituitary dysfunction. This observation aligns with the well‐established tendency for patients with AGHD to develop multiple pituitary hormone deficiencies. Although the impact of extensive pituitary dysfunction cannot be entirely excluded given the small sample size, a notable finding of this study is that the AGHD group presented significantly lower levels of both FT3 and FT4 compared with the non‐AGHD group. Notably, a postoperative decrease in multiple hormone levels is often noted among those with hypopituitarism [11], suggesting that central hypothyroidism, which was so mild as to obviate the need for levothyroxine replacement, may have been present among those exhibiting AGHD postoperatively in this study.

The main distinguishing feature of this study is that it involved ROC analysis and provided cutoff values for FT3 and FT4 14 weeks after surgery for pituitary neuroendocrine tumors to predict the diagnosis of AGHD. It is suggested that AGHD could be predicted using an FT3 cutoff value of 2.125 pg/mL and an FT4 cutoff value of 1.010 ng/dL. ROC curve analysis showed that IGF‐1 SDS had the highest AUC value. However, while FT3 and FT4 demonstrated lower AUC values compared with IGF‐1 SDS, they exhibited higher sensitivity with comparable specificity, suggesting their potential usefulness as screening parameters prior to GHST. Therefore, in patients with NF‐PitNET, it is suggested that evaluating decreases in FT3 and FT4 levels, in addition to the traditionally used IGF‐1SDS and the number of pituitary hormone deficiencies, may be useful in narrowing down the cases that require GHST.

The present study has several limitations that should be considered. First, this was a retrospective pilot study involving a small sample size, which precluded the use of multivariate analysis to account for potential confounding factors that may have influenced the results. Therefore, the findings need to be validated in a larger cohort of patients. Second, the comparison with patients who underwent the GH stimulation test (GHST), along with the greater number of male subjects in the analysis, may have introduced selection bias. Third, central hypothyroidism was evaluated postoperatively based on the use of levothyroxine. As a result, the study’s findings may be limited to cases without levothyroxine supplementation, and patients who developed hypothyroidism after the data collection were not fully excluded. Fourth, while the present study examined the presence of AGHD postoperatively, it remains unclear whether it was present preoperatively. This finding leaves the duration of AGHD in each patient uncertain and makes it difficult to determine whether surgery influences changes in GH or thyroid hormone levels. Fifth, AGHD was assessed at 12 weeks postoperatively, leaving the possibility that GH secretion might have recovered after the stimulation test. However, since no significant differences in pituitary function have been reported between 3 months and 1 year postoperatively [28], the timing of the assessment at 3 months is considered reasonable. Sixth, the timing of thyroid hormone measurement and GHST administration varied among cases. Although there were no statistical differences in the timing of thyroid hormone measurement or GHST administration between the two groups, the possibility that variations in these intervals influenced the results should be considered. Seventh, other pituitary hormones, including ACTH, cortisol, and sex hormones, may have influenced both AGHD and thyroid hormone levels. The inability to adequately examine these hormones due to the wide age and sex range and small sample size represents an additional limitation.

Despite these limitations, such as the small sample size and retrospective design, the clinical significance of our findings remains substantial. Untreated AGHD leads to progressive metabolic decline, including increased cardiovascular risks and impaired QOL [1, 2]. Performing GHST on all postoperative NF‐PitNET patients would impose an enormous burden on both patients and healthcare providers, making it unrealistic and potentially leading to delayed diagnosis or missed cases in actual clinical practice. Establishing FT3 and FT4 as convenient biochemical markers for initial screening may enable efficient determination of indications for postoperative GHST. This risk stratification approach would allow prioritization of high‐risk patients for definitive diagnosis (GHST), facilitating early initiation of GH replacement therapy and potentially reducing long‐term systemic complications associated with AGHD.

## 5. Conclusion

The individuals with AGHD after surgery for NF‐PitNET had significantly lower postoperative FT3 and FT4 levels than those with non‐AGHD. The potential utility of FT3 and FT4 levels in predicting the diagnosis of postoperative AGHD is suggested. Although FT3 and FT4 do not surpass IGF‐1 SDS as predictors of postoperative AGHD, the identification of cutoff values for FT3 and FT4 in predicting AGHD suggests their potential utility as predictive biomarkers for AGHD. It is hoped that this study may serve as a preliminary step toward the use of FT3 and FT4 levels as predictive markers for postoperative AGHD in patients with NF‐PitNET, potentially reducing the reliance on GHST through future prospective, large‐scale clinical studies.

**Nomenclature**
ACTH: Adrenocorticotropic hormones
AGHD: Adult growth hormone deficiency
ALT: Alanine transaminase
AST: Aspartate aminotransferase
AUC: Area under the curve
BMI: Body mass index
FSH: Follicle‐stimulating hormone
FT3: Free triiodothyronine
FT4: Free thyroxine
GH: Growth hormone
GHRP2: Growth hormone‐releasing peptide‐2
GHST: Growth hormone stimulation test
IGF‐1: Insulin‐like growth factor 1
IGF‐1SDS: Standard deviation score for insulin‐like growth factor 1
LH: Luteinizing hormones
NF‐PitNET: Nonfunctioning pituitary neuroendocrine tumor
ROC: Receiver operating characteristics
TSH: Thyroid‐stimulating hormone
T3: Triiodothyronine
T4: Thyroxine

## Author Contributions

Yuki Tsujimoto: study conception, data collection, data analysis, and drafting and revising the manuscript. Kenji Yamashiro: contributing to data analysis and drafting and revising the manuscript. Yuki Suzuki: data collection, data analysis, and drafting and revising the manuscript. Yui Watanabe: data collection, data analysis, and drafting and revising the manuscript. Yudo Ishii: data collection, data analysis, and drafting and revising the manuscript. Rimei Nishimura: data collection, data analysis, and drafting and revising the manuscript.

## Funding

This study received no financial support.

## Disclosure

The sponsors have no role in the publication of this article.

## Ethics Statement

This study was approved by the Ethics Committee of Jikei University School of Medicine (IRB approval number: 33‐196 [10813]) and conducted in accordance with the Declaration of Helsinki and its amendments. For this retrospective and observational study, a patient consent form was exempted from the Ethics Committee of Jikei University School of Medicine. Instead, disclosing the research contents on the website of the Jikei University School of Medicine is mandatory.

## Consent

Please see the Ethics Statement.

## Conflicts of Interest

Yuki Tsujimoto, Yuki Suzuki, Yui Watanabe, and Yudo Ishii declare no conflicts of interest. Kenji Yamashiro has received subsidies from Teijin Pharma Ltd. and Ono Pharmaceutical Co., Ltd. Rimei Nishimura has received speaker honoraria from Astellas Pharma, Nippon Boehringer Ingelheim, Eli Lilly Japan KK, Kissei Pharmaceutical, Medtronic Japan, MSD, Novo Nordisk Pharma, Sanofi KK, Abbott, and Takeda Pharmaceutical, as well as contract research fees for collaborative research with the Japan Diabetes Foundation, Taisho Pharmaceutical, Ono Pharmaceutical, Takeda Pharmaceutical, Abbott, and Böehringer Ingelheim.

## Supporting Information

Additional supporting information can be found online in the Supporting Information section.

## Supporting information


**Supporting Information** Supporting Table S1. Comparison of laboratory data between non‐AGHD patients and AGHD patients.

## Data Availability

All datasets generated and analyzed during the current study are not publicly available due to ethical reasons but are available from the corresponding author on reasonable request.
