# Supplementary material for: Utility of Postoperative Thyroid Hormone Levels in Predicting the Diagnosis of Adult Growth Hormone Deficiency in Patients Undergoing Surgery for Nonfunctioning Pituitary Neuroendocrine Tumor: A Pilot Study
Source: Int J Endocrinol. 2026 Jun 9;2026:8842344. doi: 10.1155/ije/8842344 (PMC13248110; doi:10.1155/ije/8842344)
Supplement: Supplementary file 1 — Supporting Information Supporting Table S1. Comparison of laboratory data between non‐AGHD patients and AGHD patients. [file IJE-2026-8842344-s001.docx]

**Supplementary Material**

**Supplementary Table S1.** Comparison of laboratory data between non-AGHD patients and AGHD patients.

|  | Overall (n = 35) | AGHD (n = 11) | Non-AGHD (n = 24) | *P* value |
| --- | --- | --- | --- | --- |
| Fib-4 index | 1.168 (0.892 ‒ 1.640) | 1.274 (0.676 ‒ 1.450) | 1.166 (0.904 ‒ 1.693) | 0.820 |
| AST, U/L | 21.0 (19.0 ‒ 24.0) | 23.0 (21.0 ‒ 29.0) | 20.5 (18.3 ‒ 22.0) | 0.036* |
| ALT, U/L | 16.0 (13.0 ‒ 22.0) | 21.0 (16.0 ‒ 44.0) | 15.0 (12.0 ‒ 20.0) | 0.008* |
| γ-GTP, U/L | 26.0 (19.0 ‒ 48.0) | 39.0 (21.0 ‒ 78.0) | 23.5 (17.5 ‒ 37.8) | 0.097 |
| BUN, mg/dL | 13.0 (10.0 ‒ 15.0) | 13.0 (9.0 ‒ 15.0) | 12.5 (10.0 ‒ 14.75) | 0.713 |
| Cr, mg/dL | 0.74 (0.64 ‒ 0.89) | 0.80 (0.65 ‒ 0.92) | 0.69 (0.63 ‒ 0.84) | 0.409 |
| eGFR, mL/min/m^2^ | 76.0 (66.0 ‒ 87.0) | 81.0 (63.0 ‒ 95.0) | 74.5 (66.8 ‒ 86.3) | 0.875 |
| Na, mmol/L | 143.0 (142.0 ‒ 144.0) | 143.0 (142.0 ‒ 143.0) | 143.0 (141.3 ‒ 144.0) | 0.986 |
| K, mmol/L | 4.2 (3.8 ‒ 4.3) | 4.1 (3.7 ‒ 4.2) | 4.2 (3.9 ‒ 4.4) | 0.198 |
| Cl mmol/L | 106.0 (105.0 ‒ 107.0) | 106.0 (105.0 ‒ 108.0) | 106.0 (105.0 ‒ 107.0) | 0.540 |

The data are expressed as medians (25th–75th percentiles). *P < 0.05. The Mann‒Whitney test was used for comparisons between non-AGHD patients and AGHD patients.

Abbreviations: ALT, alanine transaminase; AST, aspartate aminotransferase; BUN, Blood Urea Nitrogen; Cr, creatinine; eGFR, estimated glomerular filtration rate; Fib-4 index; Fibrosis 4 index.
